# Genome-Wide Assessment of Outer Membrane Vesicle Production in *Escherichia coli*

**DOI:** 10.1371/journal.pone.0139200

**Published:** 2015-09-25

**Authors:** Adam J. Kulp, Bo Sun, Teresa Ai, Andrew J. Manning, Nichole Orench-Rivera, Amy K. Schmid, Meta J. Kuehn

**Affiliations:** 1 Dept. of Molecular Genetics and Microbiology, Duke University Medical Center, Durham, NC, 27710, United States of America; 2 Dept. of Biology, Duke University, Durham, NC, 27708, United States of America; 3 Dept. of Biochemistry, Duke University Medical Center, Durham, NC, 27710, United States of America; 4 Duke Center for Systems Biology, Duke University, Durham, NC, 27708, United States of America; Centre National de la Recherche Scientifique, Aix-Marseille Université, FRANCE

## Abstract

The production of outer membrane vesicles by Gram-negative bacteria has been well documented; however, the mechanism behind the biogenesis of these vesicles remains unclear. Here a high-throughput experimental method and systems-scale analysis was conducted to determine vesiculation values for the whole genome knockout library of *Escherichia coli* mutant strains (Keio collection). The resultant dataset quantitatively recapitulates previously observed phenotypes and implicates nearly 150 new genes in the process of vesiculation. Gene functional and biochemical pathway analyses suggest that mutations that truncate outer membrane structures such as lipopolysaccharide and enterobacterial common antigen lead to hypervesiculation, whereas mutants in oxidative stress response pathways result in lower levels. This study expands and refines the current knowledge regarding the cellular pathways required for outer membrane vesiculation in *E*. *coli*.

## Introduction

Outer membrane vesicles (OMVs), derived from both pathogenic and commensal Gram-negative bacteria, are formed from the budding and release of the outer membrane (OM), leading to the formation of lipid-encapsulated spheres that range from 20–200 nm in diameter [1]. Periplasmic cargo is encapsulated in the lumen of OMVs, protecting it from degradative environmental factors [2, 3]. Membrane-bound cargo is found on the surface of OMVs, including outer membrane proteins (OMPs) [4], lipopolysaccharide (LPS) [5], and in some cases, quorum signaling molecules [6]. Because OMVs contain biologically active proteins, it is no surprise that they partake in a multitude of biological functions, including the delivery of toxins during infection [7–9], biofilm nucleation [10, 11], defense against antimicrobials [12], nutrient acquisition [13–15] and even DNA transfer [16, 17]. Many studies have described OMV contents and activities, but little is known about the process of OMV biogenesis. Uncovering this process will increase our understanding of a well-conserved and important bacterial pathway and may yield insights into other membrane-bounded secretion systems.

A strong body of evidence indicates that OMV production and OMV cargo loading result from regulated mechanisms and not from random occurrences or cell death [18–21]. These studies determined that the protein and lipid contents differ between the Gram-negative envelope and OMVs, suggesting that material is specifically packaged into OMVs or excluded from sites of OMV biogenesis. For example, the lipids of OMVs from *Pseudomonas aeruginosa* are mainly composed of a highly charged form of LPS known as B-band LPS, even though this molecule is only a minor component of the bacterial OM [22]. Such consistently observed enrichments rule out random membrane shedding or lysis, which would be expected to produce vesicles that are identical to the cell envelope in composition.

Studies aiming to describe the mechanism of vesiculation have led to several different models describing how Gram-negative bacteria produce OMVs. Data showing that OMV lipids differ from the lipids of the OM, such as the aforementioned report on *Pseudomonas* OMVs, have led to a model in which membrane curvature is induced by the accumulation of LPS molecules with atypical structures or charges. LPS is the major constituent of the outer leaflet of the OM of most Gram-negative bacteria. The LPS molecules themselves are not homogeneous; the length and content of the polysaccharide chain varies among the different molecules. It is proposed that subsets of these molecules may gather in patches along the OM, inducing higher degrees of membrane curvature at particular locations, either due to charge repulsion [22] or their molecular shape [23].

A second, but not necessarily mutually exclusive, model of vesiculation has been proposed involving protein determinants. In this model, vesiculation occurs at sites where proteins linking the OM and the underlying peptidoglycan layer have been excluded, disrupted or otherwise modified [24]. The OM is tethered to the peptidoglycan by an assortment of proteins that link the two layers (e.g. OmpA, Braun’s lipoprotein, Lpp, and Tol/Pal). Disruption of the Tol/Pal links, for instance, causes membrane instability and increased OM shedding [25]. Mutations have been used to show that vesiculation levels are dependent upon the proteins crosslinking the OM to the cell wall [24, 26, 27]. Supporting this model, Lpp is depleted in OMVs from *Escherichia coli* [28], and cells with deletion mutations in membrane-wall bridging proteins, such as the Tol-Pal complex and OmpA, exhibit increased vesiculation levels [29, 30]. Although mutants completely lacking these crosslinks exhibit severely compromised membrane integrity, native vesiculation occurs without causing gross membrane instability [31]. A linkage-based mechanism would therefore need to be tightly regulated.

Insights into the regulation of OMV production have resulted from genetic studies, analysis of OMV composition, and the identification of conditions affecting vesiculation. Vesiculation levels change with different growth conditions. For example, oxygen stress increases vesiculation in *P*. *aeruginosa* and *Neisseria meningitidis* [32, 33], DNA damaging antibiotics stimulate OMV production in *P*. *aeruginosa* via the SOS response [34], media composition influences vesiculation in *Lysobacter* sp. XL1 [14], and envelope stress increases vesiculation in *E*. *coli* [35]. In fact, the ability to induce vesiculation is critical for growth under some conditions [35, 36]. Based on these studies, we hypothesize that the mechanism of vesiculation has a genetic basis. Although previous studies, including a transposon mutagenesis screen, have implicated more than 20 genes in the process of vesiculation [31], no saturating screen has yet been performed, and thus the entire genetic potential of *E*. *coli* vesiculation has remained unexplored.

In this study, we assessed the genetic basis of OMV production by measuring vesiculation levels for the deletion mutant strains of the *E*. *coli* Keio library [37]. Nearly 150 mutant strains not previously known to be involved in OMV production exhibited significant vesiculation phenotypes. These mutants were used to evaluate the biological systems that govern OMV biogenesis. Specifically, systems analysis of vesiculation phenotype data in the context of gene functional ontologies and biochemical pathway datasets led to two novel predictions: (1) surface-exposed oligosaccharides negatively affect vesiculation; and (2) an intact oxidative stress response is required for wild type vesiculation levels. To our knowledge, this study provides the most comprehensive mutational analysis of bacterial vesiculation to date, and the data support a system in which both OM structures and response to abiotic stress are crucial to the mechanism of vesiculation.

## Materials and Methods

### Strains and growth conditions

Unless otherwise indicated, the Keio collection [37] was used for all mutants, and the BW25113 strain was used as the wild-type. Unless otherwise indicated, cultures were grown in Miller LB media containing 50 μg mL^-1^ kanamycin or 250 μg mL^-1^ ampicillin as necessary.

### High-throughput OMV phenotype assessment

**Fig 1:**
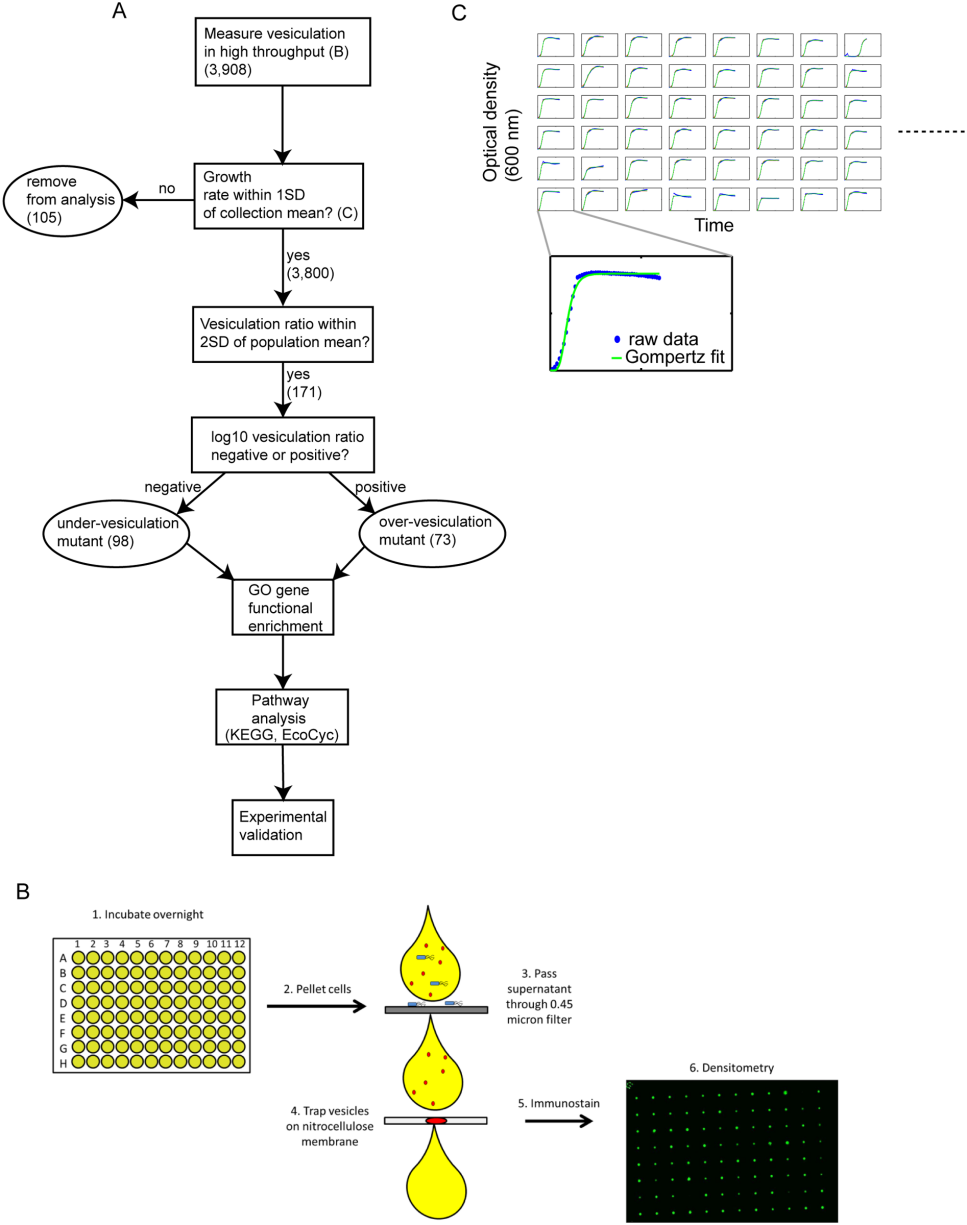
Outline of experimental and computational approaches used in this study. (A) Flowchart of experimental and computational analyses used to measure and select mutants with vesiculation phenotypes significantly different from the mean of the Keio collection (see Methods). (B) Flowchart of the high-throughput dot-blot method to determine vesiculation values. Cultures grown in 96-well plates were centrifuged to pellet cells. Using a vacuum manifold, supernatant from each culture was passed through a filter to remove remaining cells (blue) and through a nitrocellulose membrane to capture OMVs (red). The membrane was then probed using anti-LPS antibodies to detect captured OMVs, and densitometry was employed to quantify vesiculation levels. (C) Growth rates were calculated for each of the mutants tested for vesiculation to rule out lysis or poor growth. Each box represents a growth curve for one strain. The dotted line (right) represents the remaining growth curves not shown. Growth rates were calculated from raw data (blue dots) using Gompertz regression fit to the data (green line). For clarity, a growth curve for one mutant is shown in zoomed view below.

Strains from the Keio collection *E*. *coli* knock out library were inoculated into 96-well U-bottom plates (BD, Franklin Lakes, NJ, USA) containing 150 μl of LB with 50 μg mL^-1^ kanamycin and incubated at 37°C with shaking at 200 rpm overnight. To prevent evaporation, the sides of the plates were sealed with Parafilm. After the incubation, cells were pelleted by centrifugation at 1000 x g for 5 minutes. Using a vacuum manifold, 15 μl of supernatant from each well of a plate was passed through a 0.45 μm polyvinyl difluorine (PVDF) filter (Pall Scientific, Ann Arbor, MI) onto a nitrocellulose membrane (Pall Scientific). The nitrocellulose membranes were blocked in TBST (50 mM Tris-Cl, pH 7.4, 150 mM NaCl, and 1% Tween-20) containing 2% nonfat dry milk for one hour at room temperature, then incubated with polyclonal antibodies against *E*. *coli* LPS (1:1,000 in TBST; Affinity BioReagents) overnight at 4°C. The blots were then washed in TBST 6 times for 5 minutes each, followed by incubation with mouse-conjugated anti-rabbit secondary antibodies (1: 10,000 in TBST; LI-COR) for 1 hour at room temperature. The blots were again washed 5 times in TBST for 5 minutes each and once in TBS for 5 minutes. The blot was imaged using the Odyssey infrared imager (LI-COR), and the accompanying software was used to perform densitometry for the blot. This experimental procedure is summarized in Fig 1B. To account for day-to-day variations in blotting intensity, the densitometry values were normalized to the mean value of the membrane. At least two biological replicate trials were performed for each mutant strain in the library collection, and these values were averaged for each mutant. To assess the reproducibility of the assay, a subset of mutants were chosen at random for 3–5 additional biological replicate trials.

### Growth rate measurements across the Keio collection

To rule out that defects in OM vesiculation were due to impaired growth or cell lysis, the growth rate of 97% of the Keio collection mutant strains mutants was quantified. Strains were grown in Miller LB media containing 50 μg mL^-1^ kanamycin in flat-bottom 96-well plates (BD) for 18 hours (i.e. the same growth conditions as for the high throughput vesiculation assay), measuring optical density at 600 nm (OD600) every 30 minutes using a BioTek EL808 microplate reader (BioTek, USA). Growth was measured in three independent replicate trials, and the maximum growth rate was calculated using Gompertz regression [38] using custom MatLab scripts (github.com/amyschmid/GrowthFitting). Growth rates with poor fits to the Gompertz model (R^2^ ≤ 0.7) were filtered out. The remaining strains falling outside one standard deviation of the mean of growth rates for all strains across all three independent growth curves were considered to have a defective growth phenotype and excluded from subsequent analyses. The remaining 3,800 mutants with normal growth were considered in subsequent analyses. These procedures are outlined in Fig 1A and 1C.

### Data analysis

Mutants were considered to have significant over- or under-vesiculation phenotypes if they exhibited normal growth (see above) and their average vesiculation value was outside two standard deviations from the mean vesiculation value of the entire mutant collection (greater than 0.377 or less than -0.423). This cutoff was chosen to account for the per-gene variability (S1 Table). To further assess data reproducibility, additional replicates were conducted for randomly selected subsets of the library, with 272 mutants run thrice, 138 run four times, and 76 run five times. The coefficient of variation of unlogged vesiculation values (CV, mean / standard deviation) was calculated for each mutant in the collection. The mean CV for the 3,800 mutants that passed the growth filter is reported in the text. For the 171 total resultant mutants passing the significance cutoff (two standard deviations from the vesiculation mean of the population), significant enrichment (*p* ≤ 0.05) in Gene Ontology (GO) functional categories was calculated using AmiGO database from the Gene Ontology Consortium [39]. Because the GO database is not annotated for the BW25113 strain, the closest relative, MG1655, was used for all evaluations. GO categories with significant enrichment are given in Table 1, with individual gene memberships given in S5 Table. Metabolic pathway analyses were conducted by downloading KEGGxml files from Kyoto Encyclopedia of Genes and Genomes (KEGG, [40]) or pathway information from EcoCyc [41]. Visualizations shown in the Figures were produced using the KEGGScape plug-in to Cytoscape v 3.1.0 [42]. All mappings between gene name and unique identifiers were conducted using the EcoGene [43] and PortEco [44] databases.

**Table 1:** Gene Ontology (GO) functional enrichment for mutants with defects in vesiculation.

GO term (GO Biological Process ID)^a^	Background^b^	Sample^c^	*p*-value^d^
Lipopolysaccharide biosynthetic process (GO:0009103, GO:0008653)^e^	82	15	1.96E-06
ADP-L-glycero-beta-D-manno-heptose biosynthetic process (GO:0097171)	3	3	3.65E-03
Glutathione metabolic process (GO:0006749)	6	3	2.76E-02
Response to oxidative stress (GO:0006979)	75	8	4.27E-02

^a^The most specific GO categories are reported.

^b^Number of genes in the genome that are members of the GO category

^c^Number of genes identified in the analysis

^d^Significance calculated using the hypergeometric test

^e^Also GO:0046401 and GO:0009244. The related GO category with the most conservative value is reported in the table.

### Flask-grown culture OMV preparation (FCOP)

To validate the results from the high throughput method, vesiculation levels were assessed using large volume cultures and a previously described, established method [31] with some modifications, herein referred to as the FCOP method. Specifically, test and wild type control strains were grown in 250 mL cultures at 37°C overnight while shaking at 200 rpm. The *lpxL* and *lpxM* deletion mutants were grown in Lennox LB for FCOP measurements due to their sensitivity to high salt concentrations. After the incubation, a 1 mL aliquot of the culture was used to determine the number of colony forming units (CFUs) by dilution plating. Cells were removed from the culture by centrifugation at 10,000 x g for 10 minutes followed by filtering the supernatant through a 0.45 μm PVDF filter (Millipore). OMVs were pelleted from the cell-free media by centrifugation at 38,000 x g for 3 h at 4°C. The supernatant was decanted, vesicles were resuspended in the remaining supernatant, then collected by a second round of centrifugation at 100,000 x g for 1 hour at 4°C in a tabletop ultracentrifuge. The media was decanted from the tubes, and the collected OMV pellets were resuspended in 100 μl of DPBSS, salt-supplemented Dubelco’s phosphate buffered saline containing additional salts (200 mM NaCl, 8.1 mM Na_2_HPO_4_, 2.68 mM KCl, 1.47 mM KH_2_PO_4_, 0.9 mM CaCl_2_, and 0.5 mM MgCl_2_). The OMVs were quantified using the lipophilic fluorescent dye FM4-64 (Invitrogen) as described previously [31]. Vesiculation values were determined by normalizing the FM4-64 measurement to the CFUs and to the average vesiculation value of wild type control strains across at least 3 replicate trials.

## Results and Discussion

### Establishment of a high-throughput assay to quantify vesiculation recapitulates known vesiculation phenotypes and identifies novel pathways

**Fig 2:**
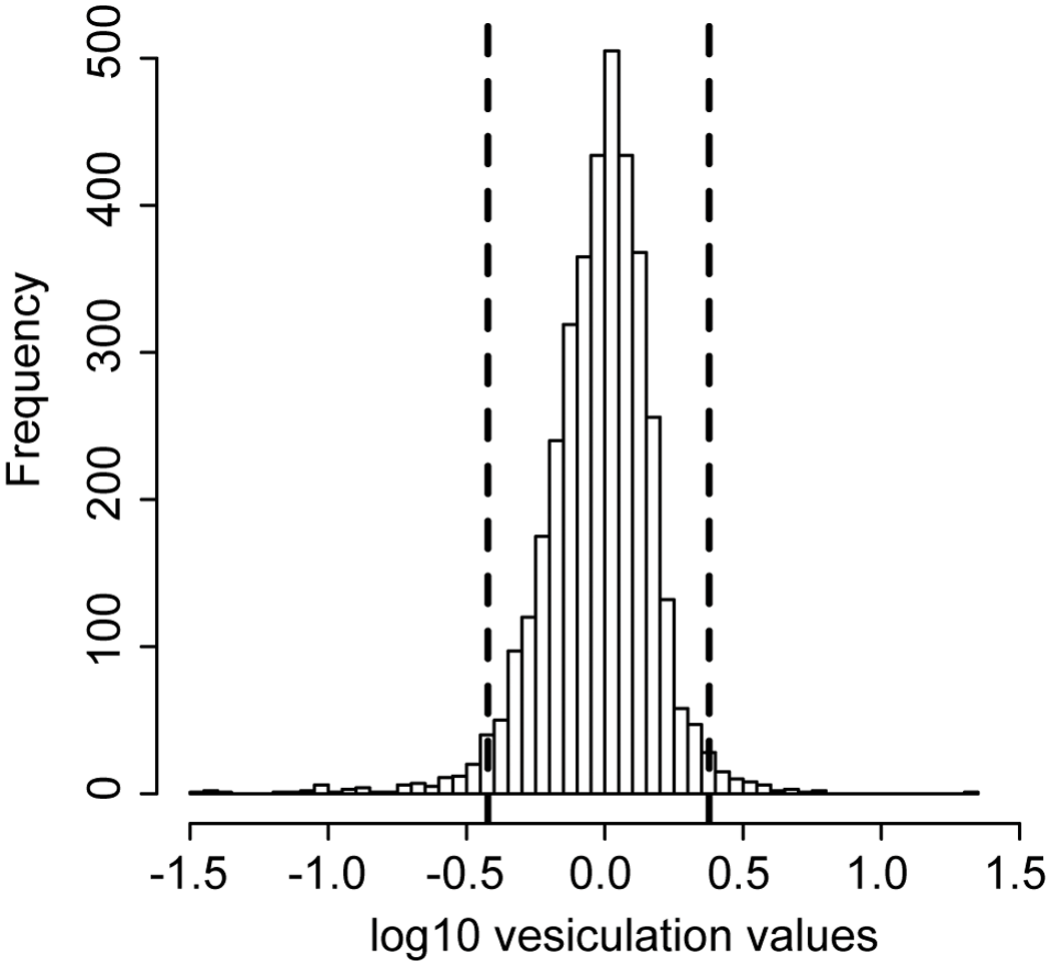
Analysis of high throughput vesiculation experiment. The range of the vesiculation scores from the high throughput method is displayed on the histogram. Mutants with log10 vesiculation scores outside two standard deviations from the mean of the distribution (vertical dotted lines) were considered in subsequent analyses.

The current methods to measure vesiculation levels accurately are based on large-scale, multistep, expensive and time-consuming assays, making genome-wide studies of vesiculation genetics infeasible. To gain global insight into the mechanism of OMV production in bacteria, we designed experimental and analytical methods to test a wide variety of mutant strains in a high-throughput, cost-effective manner (Fig 1A). Insoluble secreted extracellular material (mainly, but not exclusively vesicles) from all non-essential mutant strains representing 98% of the Keio collection grown in 96-well plates were sterile-filtered (0.45 μm) then trapped on nitrocellulose membranes. Vesicles were then quantified by high throughput immunostaining using anti-LPS antibody and densitometry (Fig 1B, Methods). Vesiculation values for the whole collection are given in S1 Table. We note here that our earlier work characterizing the membranes and cell integrity of vesiculation mutants that were detected using a similar assay showed no direct correlation between membrane integrity defects and vesicle production [31]. To rule out low vesiculation values that were due to growth impairments or high vesiculation values that were due to lysis (since the detection of LPS cannot distinguish cell fragments from vesicles), growth of strains across the collection were monitored in liquid medium in a plate reader under conditions matching those used for the vesiculation assay (Fig 1C, S2 Table). Of the initial 3,905 strains tested, 105 exhibited growth rates outside one standard deviation from the mean of the collection under the conditions tested here. These were discarded from subsequent analyses (Fig 1A, Methods, S3 Table). The vesiculation values for strains passing this growth rate filter approximated a normal distribution (mean value -0.023, Fig 2). To assess data reproducibility, additional biological replicates were conducted for randomly selected mutants in the library (Methods, S1 Table). The average coefficient of variation for vesiculation values across all replicates was 27.8%, which is expected given the biological variability of the phenotype [31]. To account for variability in the data, strains with vesiculation values outside two standard deviations from the population mean were considered to exhibit a significant vesiculation phenotype (Fig 2). This yielded 171 strains with significant vesiculation phenotypes and no growth defects (Figs 1A and 2, S4 Table). Of these, 73 mutants exhibited over-vesiculation phenotypes and 98 showed under-vesiculation phenotypes.

Previous work implicated 23 mutants in the process of vesiculation in *E*. *coli* K-12 [29–31, 45]. Of these 23, 17 showed over-vesiculation and 6 under-vesiculation phenotypes, although vesiculation was only assessed qualitatively for 3 of these mutants [29, 30, 45]. The values of 7 of the 23 mutants with previously identified vesiculation phenotypes were recapitulated here (>2 SD from the mean). Three of the 23 mutants (*tolB*, *ompR* and *degP*) previously identified as an over-vesiculating [30, 31] exhibited defective growth in the high-throughput assay conditions here and were therefore not considered for further analysis in this study. The remaining 12 mutants exhibited vesiculation values deemed not significant based on the stringent statistical criteria used here. Four of these 12 were close to the 2 SD cutoff and exhibited the same qualitative phenotype (over- or under-vesiculation). Differences in mutation types (e.g. truncations or insertions in previous studies as compared with knockouts here), different growth conditions, and different strain backgrounds may account for the discrepancies between previous and current results. Nevertheless, the assessment of the Keio collection presented in this study agreed with published data for a significant subset of the strains (hypergeometric probability *p* < 1.16 x 10^−5^).

To determine which biological functions were represented, those 171 mutants with significant vesiculation phenotypes in the high throughput assay (S4 Table) were analyzed for significant enrichment in Gene Ontology (GO) functional categories. Genes involved in the lipopolysaccharide (LPS) biosynthesis pathway and in stress response (e.g. oxidative stress, glutathione synthesis) pathways were overrepresented among vesiculation mutants (Table 1, S5 Table). Taken together, the results of the high throughput analysis of OM vesiculation assay: (a) recapitulate and quantitatively refine known vesiculation phenotypes; and (b) implicate genes and pathways in the process of vesiculation in *E*. *coli*. This high throughput method rapidly, quantitatively, and reliably identifies vesiculation phenotypes for the entire Keio collection, representing a rich and novel dataset for exploration.

### An intact stress response is required for vesiculation

Significant enrichment was detected in glutathione biosynthesis and oxidative stress response GO categories. Whereas vesiculation has previously been linked to oxidative stress for other species [33, 46], a relationship between oxidative stress and vesiculation has not been previously identified for *E*. *coli*. The association between glutathione synthesis and vesicle production was novel. Of the 81 genes in the genome included in these two categories, 11 mutants tested here exhibited significant vesiculation defects (Table 1). The majority of these mutants (8 of 11) exhibited under-vesiculation phenotypes (S4 and S5 Tables). Specifically, under-vesiculation was detected for mutants in genes whose products are required for transcriptional regulation of oxidative stress response (*oxyR*) and neutralization of oxidants (e.g. catalase-encoding *katG*) as well as glutathione biosynthesis genes. OxyR is a transcriptional activator of three of the genes with under-vesiculation phenotypes (*katG*, *gor*, *mntH* [47, 48]) suggesting a regulatory link between oxidative stress and OMV production. These findings are consistent with previous work suggesting that oxidative stress stimulates OMV production in *Pseudomonas* [46]. However, this study newly implicates specific oxidative stress production genes and potential transcription regulators in *E*. *coli* OMV production. Together these results suggest that an intact oxidative stress response is required for wild type OMV production levels in *E*. *coli*.

### Vesiculation levels increase in mutants that truncate LPS

**Fig 3:**
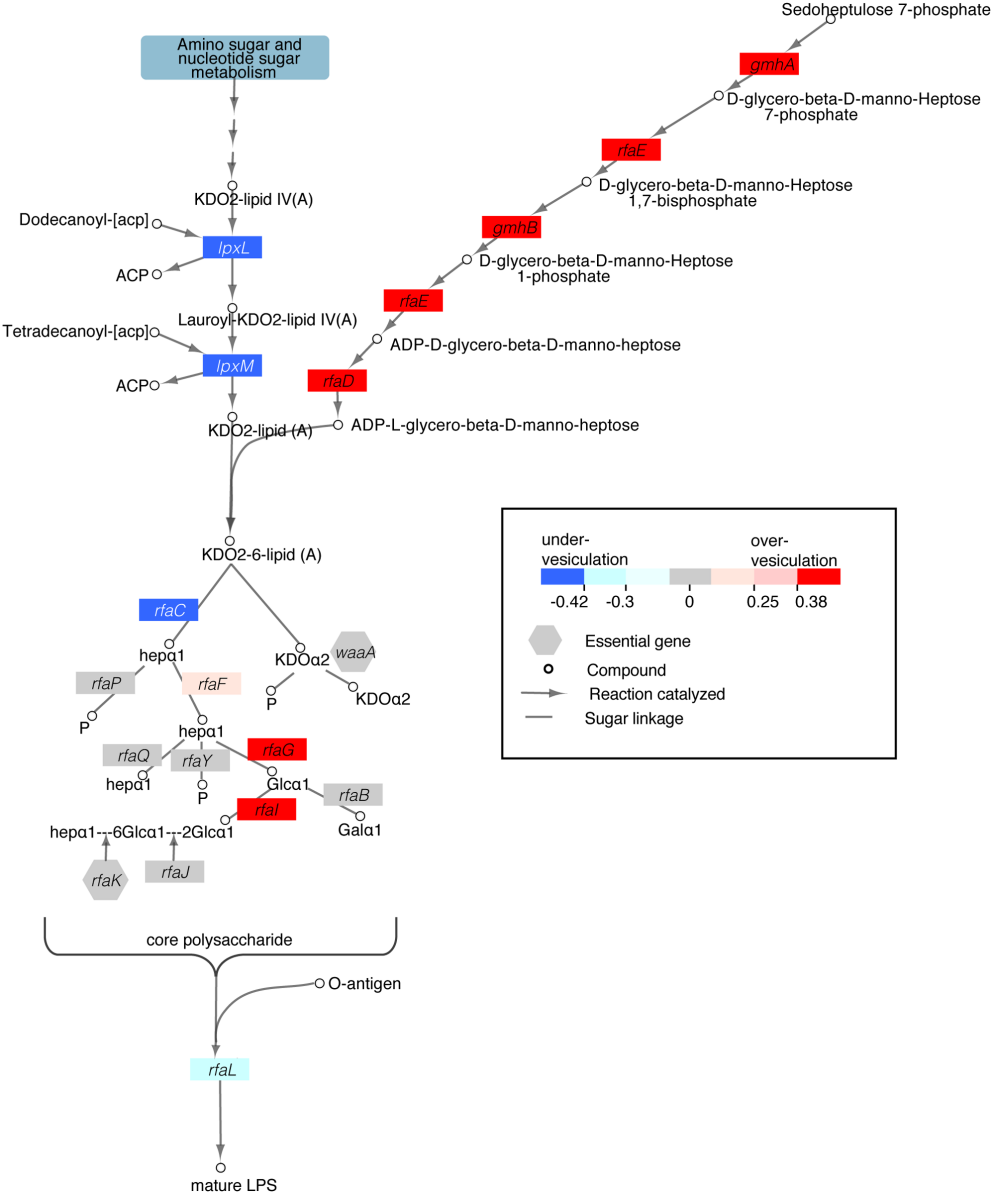
Biosynthesis of immature LPS alters vesiculation. Vesiculation phenotypes of the LPS core sugar biosynthesis pathway are depicted in the pathway diagram. Colored boxes represent vesiculation values for mutants of enzyme-coding genes at each step of the pathway. The vesiculation score for the mutants of each respective enzyme are indicated by the color of the box (see legend). Pathway adapted from KEGG [40].

An unprecedented number of genes encoding enzymes in the LPS biosynthesis pathway were implicated in OMV production (Table 1, S5 Table). Excluding the O-antigen synthesis genes that are inactive in K-12 strains, there are 81 genes listed in the Gene Ontology (GO) category for LPS biosynthesis. Of these 81 genes, deletions of 15 of the non-essential LPS biosynthesis genes resulted in significant vesiculation phenotypes, with 10 exhibiting over-vesiculation and 5 under-vesiculation (Fig 3, S4 and S5 Tables). When complemented by the expression of *rfaD in trans*, the hypervesiculation phenotype of a representative mutant Δ*rfaD* was reduced, as expected, although not to wild type levels, likely due to the nonnative expression levels of the gene (S1 Fig). Note that three of these 15 genes participate in enterobacterial common antigen (ECA) biosynthesis (*rffC*, *rffA*, *wzzE*), which are discussed in more detail below. Interestingly, when integrated with LPS metabolic pathway information, we observed that the majority of the 15 genes encode enzymes required to synthesize the sugar chain built upon the KDO-lipid (Fig 3), with mutants in the majority of these genes increasing vesiculation.

These findings are in agreement with prior studies in *E*. *coli* [31] as well as other studies in *P*. *aeruginosa* [22, 49] and *Porphyromonas gingivalis* [23], with the polysaccharide chain and LPS structure being implicated in the process of OM vesicle production. However, data presented here extend and refine these previous observations by identifying the full suite of non-essential genes related to the LPS pathway, which are required for wild type vesiculation levels. These genes encode enzymes involved in constructing the core sugar chain as well as two of the acyltransferase genes. Mutations to these genes result in LPS molecules with truncated polysaccharide chains or lipid cores that lack one of the six acyl chains [50](Fig 3). Taken together, these data are consistent with the hypothesis that the structure of LPS affects the vesiculation levels of *E*. *coli*, with incomplete structures yielding more OMVs.

### Vesiculation levels are inversely proportional to ECA chain length

**Fig 4:**
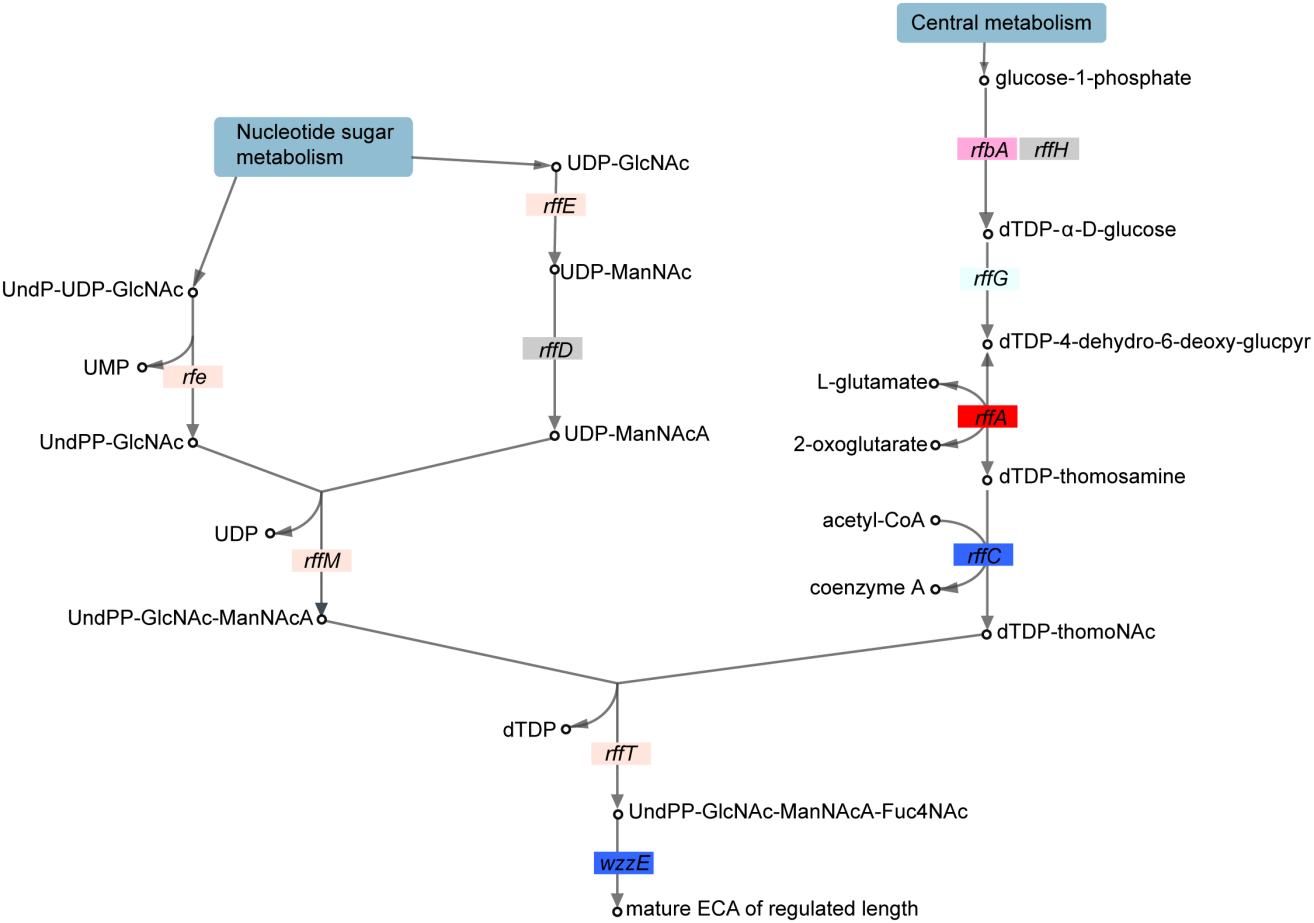
ECA biosynthesis regulates vesiculation. The network for ECA biosynthesis is shown. Colors, arrows, boxes, and dots are as in Fig 3. Pathway adapted from EcoCyc [41]. Abbreviations for sugar intermediates are as follows: UDP-GlcNAc, UDP-N-acetyl-glucosamine; UDP-ManNAc, UDP-N-acetyl-mannosamine; UDP-ManNAcA, UDP-N-acetyl-mannosaminouronate; UndP-UDP-GlcNAc, undecaprenyl phosphate-UDP-N-acetyl-glucosamine; glucpyr, glucopyranose; dTDP-thomoNAc, dTDP-N-acetylthomosamine.

Among the mutants with significant vesiculation phenotypes represented in the GO category entitled “LPS biosynthesis”, Δ*rffA (wecE)*, Δ*rffC (wecD)*, and Δ*wzzE (o349)* were detected. These genes are known to play additional roles in construction of the membrane-bound polysaccharide enterobacterial common antigen (ECA; [51]). Upon integration of these data with the currently known ECA biosynthesis pathway, we observed significant over-vesiculation in the case of the Δ*rffA* mutant and under-vesiculation for Δ*wzzE* (Fig 4). The phenotype of Δ*rffA* was restored to wild-type level by complementation with *rffA* expressed *in trans*. The expression of *wzzE in trans* increased the level of vesicle production of the Δ*wzzE* mutant in the complementation assay (S1 Fig). WzzE is the ECA chain length regulator [52], whereas RffA and C catalyze the assembly and elongation of the polysaccharide chain [53]. Several other mutants in enzyme-coding genes for this pathway also exhibited over-vesiculation phenotypes that were close to but missed the two standard deviation cutoff (Fig 4). Although the structure and biosynthesis of ECA are understood, little is known about the function of this molecule [52–54]. ECA forms a capsule around *E*. *coli* either in free cyclic form or bound to the bacterial surface through covalent bonds with peptidoglycan or LPS [55]. Recently, ECA biosynthesis has also been shown to influence vesiculation in *Serratia marcescens* [56], supporting our data (Fig 4). Here we have expanded the understanding of which enzyme-coding genes in the ECA pathway are required for vesiculation. Taken together, the vesiculation data are consistent with the hypothesis that ECA structure in *E*. *coli* is associated with vesiculation.

### Hypothesis testing and validation of high throughput data

**Fig 5:**
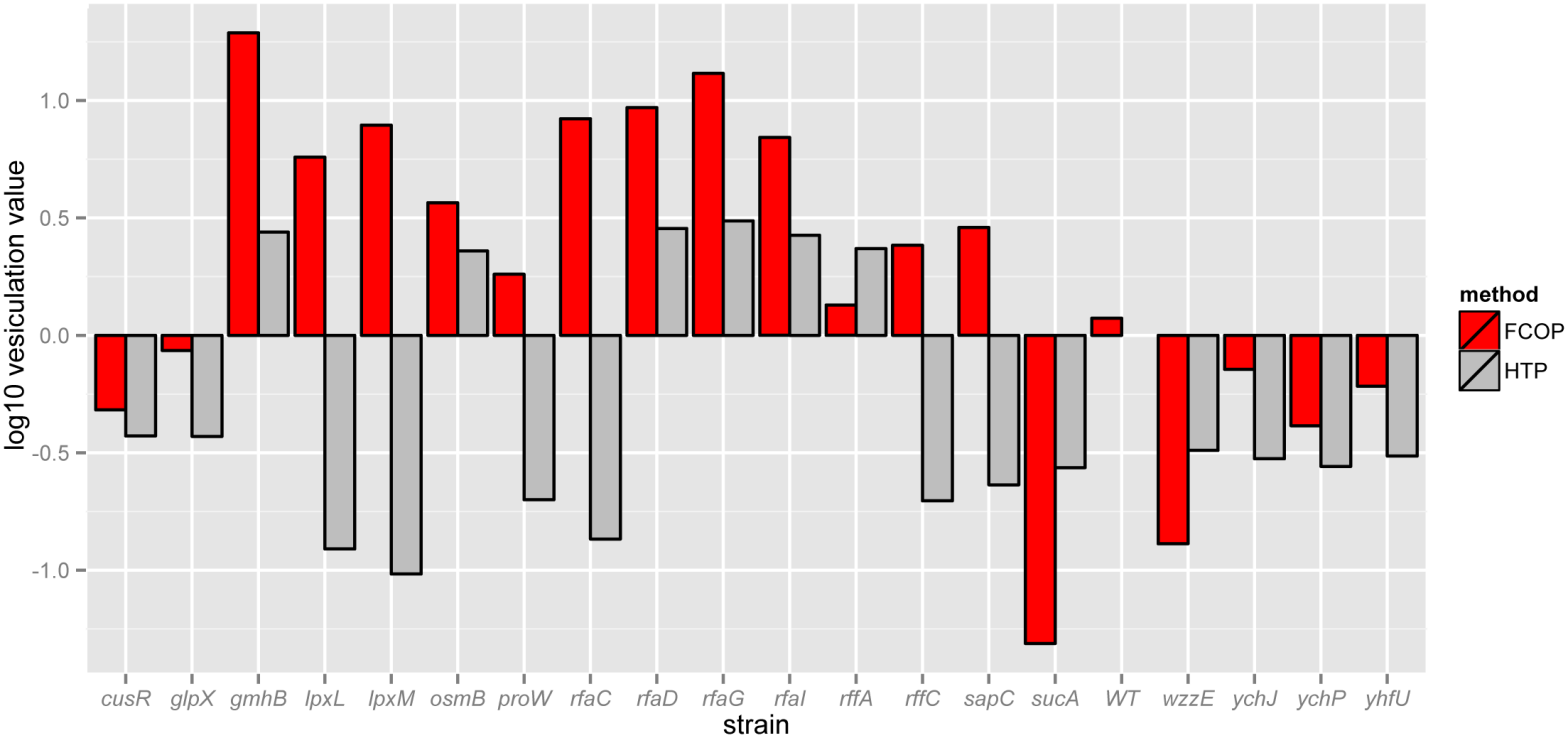
Validation and hypothesis testing. Vesiculation levels were tested using the established FCOP method for selected mutants with vesiculation values significantly different from the mean according to the high throughput dot-blot method. Bar graph compares vesiculation values as measured by the whole genome high throughput screen (HTP) vs. large-scale vesicle preparation (FCOP). Each bar represents the average of at least 3 biological replicate trials. These mean values and standard error are given in S4 Table.

To test the hypothesis that immature ECA and LPS chain length promote vesiculation and to validate our high throughput data, 10 individual mutants of the ECA and LPS biosynthesis pathways as well as 10 other randomly chosen mutant strains were analyzed using a flask-grown culture OMV preparation (FCOP) method. FCOP is a higher culture volume assay that uses a membrane dye rather than immunostaining to quantify vesicles, providing an orthogonal metric to quantify vesicle production (Methods, [31]). Good correlation between the high throughput and FCOP methods was detected (Cp = 0.40; *p* < 0.04). The direction of vesiculation (over- vs. under-vesiculation) corresponded for the majority of strains (14 of 20) across methods (Fig 5). In some cases where the phenotypes did not correspond (e.g. Δ*lpxL*, Δ*lpxM*, Δ*rffC*), these results could be linked to discrepancies in growth conditions between the two assays, since some of these mutants are known to exhibit growth defects and/or lysis in large volume cultures, which was not observed in the 96-well plate conditions (S2 Table, [57]). Nevertheless, vesiculation phenotypes for a significant number of mutants in genes encoding chain-elongating enzymes in both LPS and ECA pathways as well as other randomly chosen genes were recapitulated with FCOP (Fig 5, hypergeometric probability *p* < 1.63 x 10^−16^).

Whether LPS and ECA play a direct or indirect role in vesiculation remains as an important question for follow-up study. For example, the impact of producing immature versions of these structures on the physical properties of the cell surface may influence vesiculation by altering fluidity, the propensity to curve, or the ability to bind peripheral proteins that influence vesiculation ([23, 58–60]; K. Bonnington and M.J. Kuehn, unpublished observations). Alternatively, biosynthetic intermediate cell surface structures that accumulate in the mutants could affect envelope homeostasis and alter transcriptional or other regulatory pathways [26, 51, 61–64], leading to differences in vesiculation. Specifically, disruptions to either the LPS or ECA biosynthesis pathways activate the σ^E^ envelope stress response pathway [51, 65], which has been linked to vesiculation in *E*. *coli* and other Gram-negative bacteria [31, 35, 66, 67].

In summary, these validation tests: (a) support the validity of the high-throughput method in identifying true positive vesiculation phenotypes; and (b) provide further support for a novel hypothesis that truncated ECA and LPS chain lengths promote vesiculation in *E*. *coli*.

## Conclusion

Here we have developed a high-throughput method to measure vesiculation phenotypes for mutants in the Keio collection in a quantitative, reproducible, and cost-effective manner. The results of this study reveal that LPS and ECA structures play roles in vesiculation in *E*. *coli*. In addition, this study shows an association of stress response pathways with vesiculation. Overall, these findings suggest that wild-type levels of OMV production depend on mature bacterial surface structures and unstimulated compatibility with the extrinsic environment, whereas deviations from OMV production can be caused by perturbations in cell surface components or by stimulation of bacterial stress responses. Overall, this study has implicated nearly 150 new genes influencing process of vesiculation, suggesting that OMV production can be stimulated or inhibited through a multi-faceted mechanism.

## Supporting Information

S1 Fig Flask-grown culture OMV preparation (FCOP) data for complemented representative mutant phenotypes. (PDF)

S1 Table Outer membrane vesiculation values for 3,908 mutants in the *E*. *coli* Keio collection. (XLSX)

S2 Table Growth rate data for Keio collection mutants. (XLSX)

S3 Table Gene names of mutants filtered out of the analysis due to poor growth under conditions tested in this study. (XLSX)

S4 Table Mutants in Keio collection with normal growth rates and outer membrane vesiculation values two standard deviations from the mean of the collection. (XLSX)

S5 Table Genes comprising enriched GO categories listed in main text Table 1. (XLSX)
