# Supplementary material for: Genome-Wide Assessment of Outer Membrane Vesicle Production in Escherichia coli
Source: PLoS One. 2015 Sep 25;10(9):e0139200. doi: 10.1371/journal.pone.0139200 (PMC4583269; doi:10.1371/journal.pone.0139200)
Supplement: S1 Fig — (PDF) [file pone.0139200.s001.pdf]

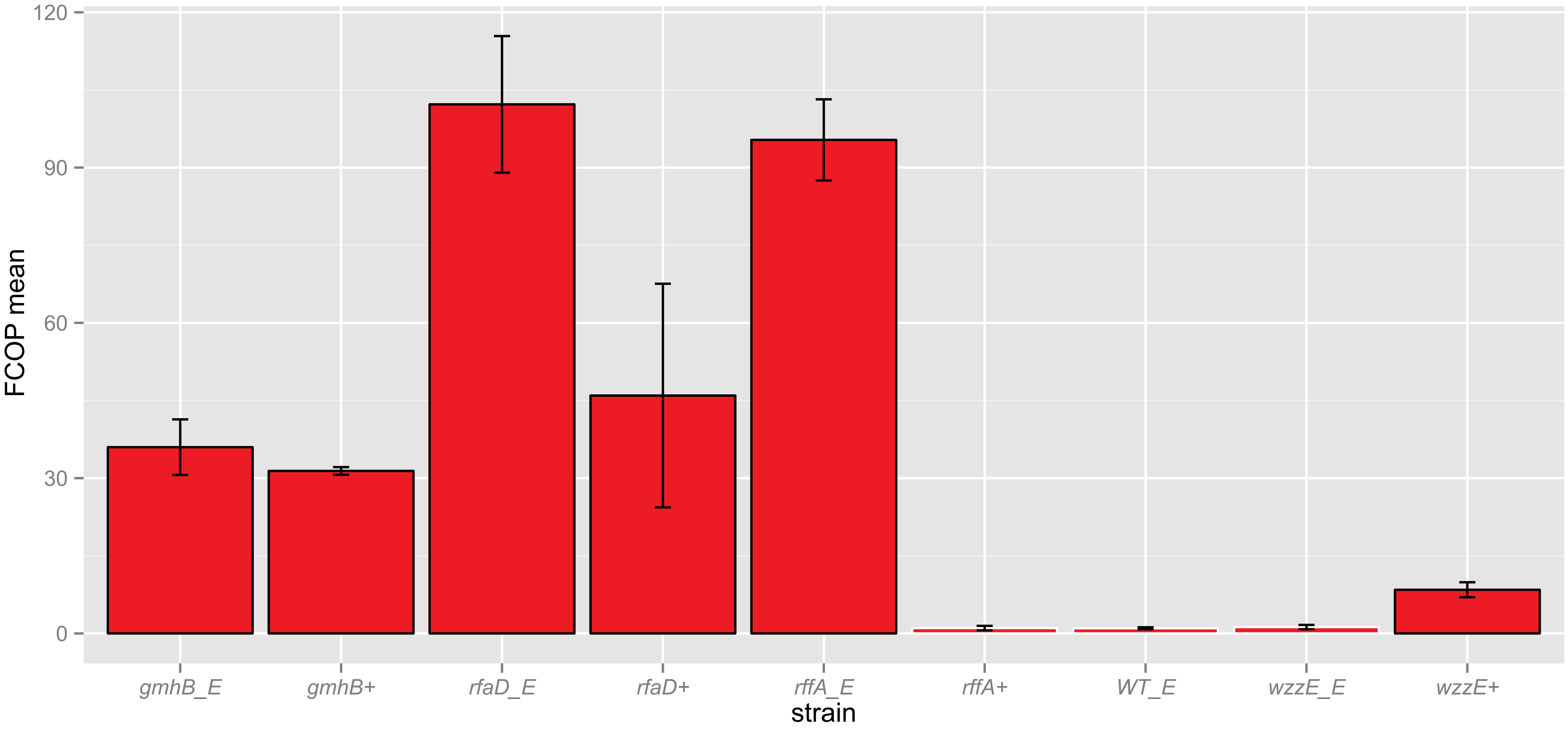

Supplementary Figure S1. Complementation of vesiculation mutants. Each bar represents the average of at least three indpendent trials of FCOP vesiculation measurements normalized to wild type vesiculation and cell numbers. "_E", mutant with empty vector; "+", mutant with complementing gene expressed in trans from a plasmid. Plasmids were obtained from the ASKA collection (Kitagawa et al., 2006, DNA Research, 12(5): 291-299). Plasmid-bearing strains were grown according to recommendations in Kitagawa et al., except that expression was not induced with IPTG.
